# Post-ictal, lateralized hyperkinetic motor behavior^☆^^☆☆^

**DOI:** 10.1016/j.ebcr.2012.10.002

**Published:** 2012-11-07

**Authors:** Brian Beck, Gregory Youngnam Chang

**Affiliations:** Department of Neurology, Barrow Neurological Institute, Phoenix, AZ, USA

**Keywords:** Unilateral hyperkinetic motor behavior, Post-ictal

## Abstract

Recognition of a transient, focal neurologic dysfunction after a seizure is important when evaluating patients with idiopathic epilepsy. Todd's palsy, a transient focal weakness after a seizure, is a highly accurate clinical sign indicative of a contralateral, cerebral epileptic focus. In contrast, a transient, lateralized hyperkinetic motor behavior from a contralateral, hemispheric ictal focus has not been emphasized as a localizing clinical sign.

The following case demonstrates that transient hyperkinetic behavior occurs as a post-ictal phenomenon and may have a localizing value, as in Todd's palsy.

## 1 Case report

**Fig. 1:**
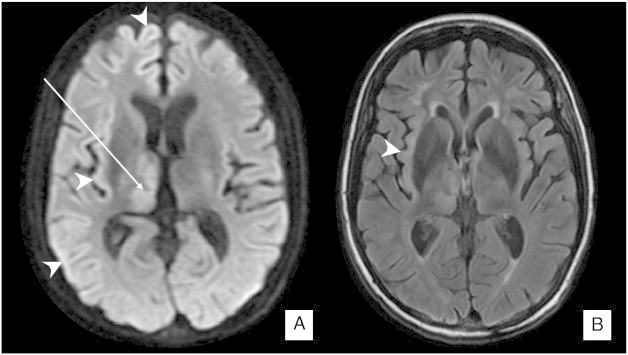
A. DWI obtained 5 days after admission reveals a subtle, increased signal over the large area of the right cortical ribbon, most prominent over the hippocampus (arrow). Right thalamic involvement is more prominent (arrow). The ADC map reveals a subtle low signal consistent with cytotoxic edema (not shown). B. FLAIR sequence on the same day reveals a higher signal mainly over the right hippocampus and ipsilateral thalamus.

A 46-year-old woman with chronic seizure disorder was brought to the emergency room after 3 episodes of generalized tonic-clonic seizures. She was given intravenous lorazepam, a loading dose of fosphenytoin, and intubated. The follow-up EEG that was obtained several hours later disclosed right-sided, semi-rhythmical, large-amplitude spikes consistent with partial status epilepticus. The examination next day disclosed an awake, intubated patient who followed a simple command of “show me your right thumb” without any difficulty. There was no evidence of gaze deviation or nystagmus. There was no abnormal facial movement, but the left arm demonstrated continuous, semi-purposeful movements with frequent grabbing of the bedcover but without tonic–clonic or rhythmical components. When instructed not to move the left hand, she mouthed “I am trying,” but within seconds, her movements returned. Similarly, the left foot demonstrated occasional, semi-purposeful ankle movements. Two days later, now extubated, she fed herself with the right hand but continued with left lateralized hyperkinetic motor behavior (LHMB). When she was asked to show the left little finger, her response was delayed but appropriate. When she was asked to grab the pen that was already placed in her left palm, she had poor control and was unable to hold the pen more than a second. There was no obvious left-sided hemineglect, feeling of alieness or sensory loss. The follow-up EEG demonstrated background rhythm slowing over the right hemisphere but no epileptiform activity. The new brain MRI demonstrated patchy increased signal over the right cortical ribbon, along with right thalamic hyperintensity (Figs. 1A and B). Five days after the onset of the right hemispheric, partial status epilepticus, the left-sided hyperkinetic motor movements resolved with subtle, left wrist extensor weakness.

## 2 Discussion

Non-lateralized, hyperkinetic motor behavior is relatively common as part of a transient, global confusional state after a seizure. In this context, hand movements are purposeful, albeit non goal-directed, yet are under full voluntary control. Lateralized hyperkinetic motor behavior, as seen in this case, is a continuous, compulsive, abnormal motor behavior, which is semi-purposeful, under poor voluntary control, and clinically can be suspected by a repetitive manipulation of nearby objects such as a bedcover, while independent of attentiveness, visual input, or tactile stimulation. Other movement disorders such as alien hand phenomenon, ‘reflexive’ movement such as grasping with inability to release, and utilization behavior from frontal or thalamic lesions may look similar but can easily be excluded on careful clinical observation.

Although unilateral on clinical manifestation, LHMB may require initially bilateral cerebral dysfunction. Chee et al. [1], in a study based on video monitoring, noted similar lateralized hand fumbling movements as an ictal phenomenon. Almost all of their 8 cases started initially with bilateral hand fumbling that evolved into predominant unilateral involvement. Overall, a transient LHMB is more commonly seen in the context of an acute unilateral large hemispheric stroke. Limbs, ipsilateral to the acute infarction, demonstrate continuous, semi-purposeful movement with contralateral limbs typically hemiplegic. Transient, physiologic cortical suppression of the non-infarcted hemisphere via transcallosal diaschisis has been hypothesized to explain this clinical phenomenon [2]. The presence of LHMB in case of a bi-hemispheric stroke further supports this hypothesis [3]. Similarly, our case initially presented in generalized status epilepticus that evolved into right hemispheric lateralization. We cannot completely exclude the LHMB as a residual ongoing focal epileptic phenomenon; however, persistent movements for 4 days accompanied by complete suppression during sleep suggest that ictal manifestation is unlikely. To the best of our knowledge, LHMB has not been previously reported as a post-ictal phenomenon. Accompanying drowsiness, confused-agitated state, and concomitant Todd's paresis may have contributed to a lack of clinical recognition.

Brain MRI revealed a transient, subtle right cortical ribbon and a prominent right thalamic signal on DWI and/or FLAIR sequences (Fig. 1), which all resolved 2 weeks later. The transient nature of these signals – crossing of vascular boundaries and simultaneous thalamic involvement – suggests that this represents post-ictal-associated MRI changes as previously described [4].

## 3 Conclusion

Transient LHMB may be seen as a post-ictal phenomenon from a contralateral cerebral ictal focus. Further clinico-radiologic correlation with physiologic studies will be necessary to delineate the topographic area that is responsible to manifest this “paradoxical” Todd's palsy sign. Its ultimate usefulness in pre-surgical evaluation, its role in thalamic involvement as seen in this case, and its physiologic relationship with Todd's palsy remain to be clarified.
